# Aptamer-Functionalized Ce^4+^-Ion-Modified C-Dots: Peroxidase Mimicking Aptananozymes for the Oxidation of Dopamine and Cytotoxic Effects toward Cancer Cells

**DOI:** 10.1021/acsami.2c16199

**Published:** 2022-12-07

**Authors:** Yu Ouyang, Michael Fadeev, Pu Zhang, Raanan Carmieli, Yang Sung Sohn, Ola Karmi, Yunlong Qin, Xinghua Chen, Rachel Nechushtai, Itamar Willner

**Affiliations:** † The Institute of Chemistry, The Hebrew University of Jerusalem, Jerusalem 91904, Israel; ‡ Department of Chemical Research Support, Weizmann Institute of Science, Rehovot 76100, Israel; § Institute of Life Science, The Hebrew University of Jerusalem, Jerusalem 91904, Israel

**Keywords:** nanozyme, aptamer, peroxidase, reactive oxygen species, chemodynamic cancer therapy

## Abstract

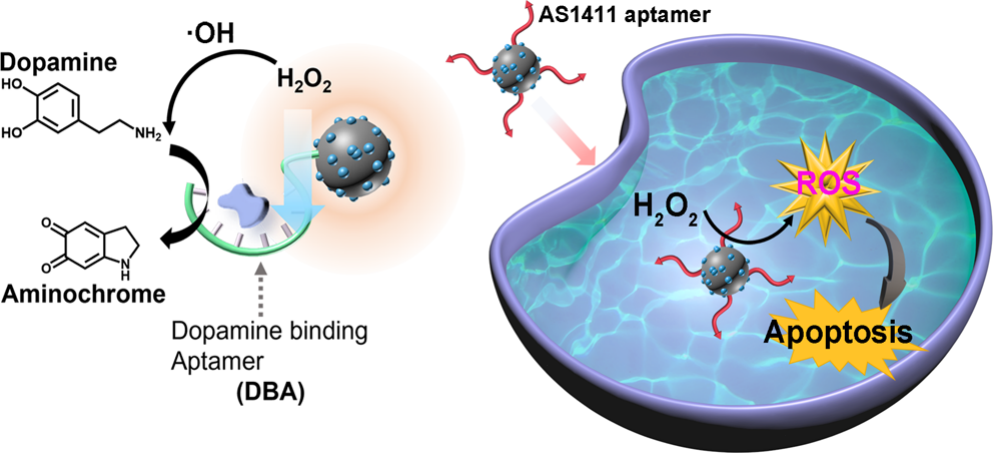

Aptamer-functionalized Ce^4+^-ion-modified C-dots act as catalytic hybrid systems, aptananozymes, catalyzing the H_2_O_2_ oxidation of dopamine. A series of aptananozymes functionalized with different configurations of the dopamine binding aptamer, DBA, are introduced. All aptananozymes reveal substantially enhanced catalytic activities as compared to the separated Ce^4+^-ion-modified C-dots and aptamer constituents, and structure–catalytic functions between the structure and binding modes of the aptamers linked to the C-dots are demonstrated. The enhanced catalytic functions of the aptananozymes are attributed to the aptamer-induced concentration of the reaction substrates in spatial proximity to the Ce^4+^-ion-modified C-dots catalytic sites. The oxidation processes driven by the Ce^4+^-ion-modified C-dots involve the formation of reactive oxygen species (^•^OH radicals). Accordingly, Ce^4+^-ion-modified C-dots with the AS1411 aptamer or MUC1 aptamer, recognizing specific biomarkers associated with cancer cells, are employed as targeted catalytic agents for chemodynamic treatment of cancer cells. Treatment of MDA-MB-231 breast cancer cells and MCF-10A epithelial breast cells, as control, with the AS1411 aptamer- or MUC1 aptamer-modified Ce^4+^-ion-modified C-dots reveals selective cytotoxicity toward the cancer cells. In vivo experiments reveal that the aptamer-functionalized nanoparticles inhibit MDA-MB-231 tumor growth.

## Introduction

Inorganic metal, metal oxide, core–shell metal composite, and carbon-based or organic-based polymer nanoparticles find growing interest as catalytic materials mimicking enzyme activities, “nanozymes”.^1−5^ Metal nanoparticles, such as Au,^6−8^ Ag,^9^ or Pt;^10,11^ metal oxide nanoparticles, such as Fe_3_O_4_,^12−14^ V_2_O_5_,^15,16^ CeO_2_,^17^ or MO_3_;^18^ carbon nanomaterials, such as metal-ion modified C-dots,^19^ graphene oxide,^20,21^ or carbon nitride particles;^19^ core–shell metallic composites, such as Ag@Cu nanoparticles;^22^ and metal–ligand clustered nanoparticles, such as Prussian blue derivatives,^23,24^ demonstrate nanozyme activities catalyzing diverse chemical processes. In addition, metal-ion-modified metal–organic framework nanoparticles (NMOFs),^25−28^ such as Cu^2+^-ion-functionalized UiO-66^25^ or Au^3+^-modified NMOFs,^29^ and organic composites, such as polydopamine^30^ or melanin particles,^31^ reveal catalytic nanozyme activities. Diverse nanozyme-catalyzed reactions mimicking native enzyme activities were reported, including peroxidase-like activities,^21,25,32^ for example, oxidation of tetramethyl benzidine or of Amplex-Red by H_2_O_2_, oxidation of dopamine or NAD(P)H by H_2_O_2_, or generation of chemiluminescence by the catalyzed oxidation of luminol by H_2_O_2_. Also, oxidase-like activities, such as aerobic oxidation of glucose;^33,34^ hydrolase-like activities, such as hydrolysis of urea;^35^ or phosphatase-like activities^36^ were demonstrated by nanozymes. Different applications of nanoparticle nanozymes were reported, including their use as amplifying labels for electrochemical^37,38^ or optical sensing,^39−41^ biomedical applications such as imaging^42−44^ and cancer therapies,^45−48^ and treatment of other diseases such as Alzheimer’s,^49,50^ Parkinson’s,^51^ or cardiovascular diseases.^52^ Nanozymes were also applied as antibacterial^53^ and wound-healing agents^54,55^ and active catalysts for the degradation of pollutants.^56,57^

While nanozymes reveal enhanced stabilities as compared to native enzymes, their turnover rates are substantially lower as compared to enzymes, mainly due to the lack of mechanisms to concentrate the substrate at the catalytic interfaces (molarity effect), and the lack of stereoselectivity or chiroselectivity in their chemical transformations. Different approaches to overcome these limitations were suggested, including the functionalization of the nanozymes with chiral receptor binding sites, for example, β-cyclodextrins^19^ or the surface modification of the particles with imprinted polymer films.^32^ An alternative approach has involved the functionalization of nanozymes with aptamer binding tethers acting as chiral- and substrate-selective binding sites. These hybrid nanozymes were termed “aptananozymes” and exemplified with the synthesis of Cu^2+^-ion-modified C-dots functionalized with the anti-dopamine aptamer or anti-tyrosinamide aptamer.^58^ Tethering a set of respective aptamers to the Cu^2+^-ion-modified C-dots by linking directly the 3′- or 5′-ends of the aptamers to the C-dots or through variable lengths of spacer bridges resulted in aptananozymes that catalyzed the oxidation of dopamine to aminochrome in the presence of H_2_O_2_, or the insertion of oxygen into the Ar–H bond of l-tyrosinamide to form the respective catechol product that was subsequently oxidized to amidodopachrome, in the presence of a H_2_O_2_/ascorbic acid mixture. The sets of aptamer-functionalized nanozymes, aptananozymes, revealed enhanced catalytic activities as compared to the separated nanozyme/aptamer constituents, and structure–function relationships were controlled by the structure of the aptamer and their modes of conjugation to the nanozymes. The enhanced activities of the aptananozymes were attributed to the aptamer-induced concentration of the substrate in spatial proximity to the catalytic nanozyme, in analogy to the active site structures of native enzymes. The assembly of the Cu^2+^-ion-modified C-dots aptananozymes was, however, a single example of this class of hybrid catalysts. Broadening this class of catalysts to other aptananozyme compositions and the introduction of aptananozymes are essential to demonstrate the utility of these nanomaterials.

Realizing that CeO_2_ demonstrated effective peroxidase and oxidase activities,^59,60^ we attempted to probe the Ce^4+^-ion-modified C-dots as a potential peroxidase/oxidase nanozyme. While the Ce^4+^-ion-modified C-dots did not show oxidase activities, under aerobic conditions, they demonstrated effective peroxidase functions and were thus selected as a potential nanozyme for developing aptananozymes. Here, we wish to report on the synthesis of Ce^4+^-ion-modified C-dots functionalized with different configurations of the dopamine binding aptamer (DBA). The different aptamer-functionalized C-dots reveal peroxidase-like catalytic activities and catalyze the H_2_O_2_-driven catalyzed oxidation of dopamine to aminochrome. Structure–catalytic function relationships are elucidated within the set of aptananozymes. In addition, the chiroselective affinity binding of D/l-DOPA to the dBA aptamer associated with the C-dots leads to the chiroselective oxidation of D/l-DOPA to dopachrome. Mechanistic studies reveal that the oxidation of the catechol substrate to the quinoid products proceeds via the Ce^4+^-ion-modified C-dot- catalyzed dissociation of H_2_O_2_ to the reactive hydroxyl radical, ^•^OH, as reactive oxygen species (ROS) products. The availability of H_2_O_2_ in cancer cells^61−64^ and the success to modify the Ce^4+^-ion-modified C-dots with aptamer tethers are then used to functionalize the Ce^4+^-ion-modified C-dots with cancer cell-specific biomarkers, for example, AS1411^65,66^ or MUC1^67,68^ aptamer, to yield targeted aptananozymes for chemodynamic treatment of cancer cells. Effective and selective formation of ^•^OH species in MDA-MB-231 breast cancer cells is demonstrated, leading to selective cytotoxicity toward the cancer cells. The chemodynamic selective cytotoxicity of the aptamer-functionalized Ce^4+^-ion-modified C-dots is examined by in vitro cell experiments followed by in vivo experiments in mice.

## Results and Discussion

**Figure 1:**
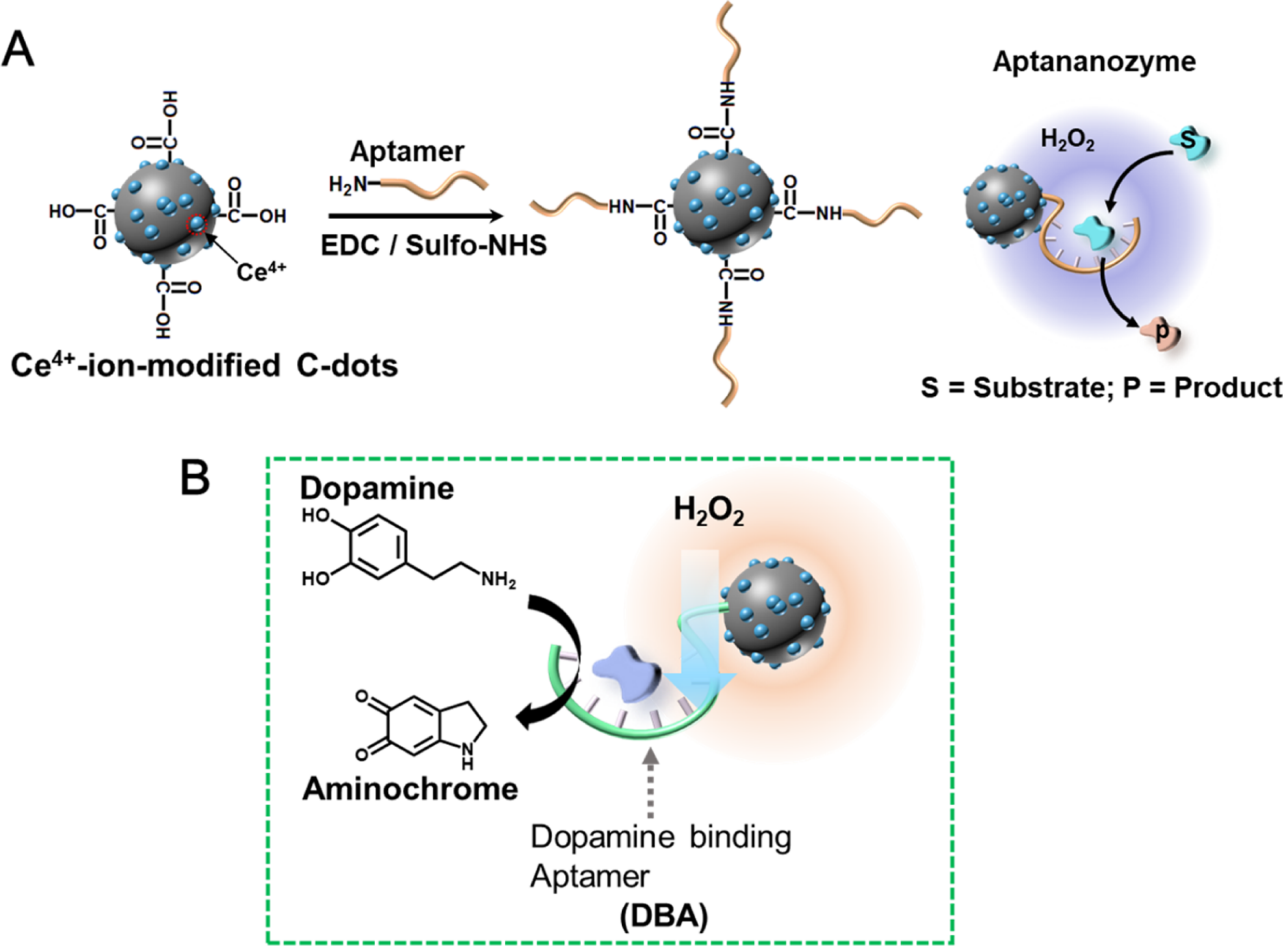
(A) Synthesis of the DBA-functionalized Ce^4+^-ion-modified C-dots, aptananozymes. (B) Schematic application of the aptananozyme assemblies toward the DBA-aptananozyme-catalyzed oxidation of dopamine to aminochrome by H_2_O_2_.

The C-dots (10 nm, Figure S1) include carboxylic acid and amine residues on their surfaces were prepared by a microwave treatment of a mixture of citric acid and urea, according to the reported procedure.^69^ The C-dots were treated with ammonium cerium(IV) nitrate to associate the Ce^4+^-ions on the C-dots via coordination interactions, resulting in Ce^4+^-ion-modified C-dots. The inductively coupled plasma mass spectrometry measurement indicated Ce^4+^-ions on the C-dots particle, corresponding to coverage of 300 ± 5 μg per mg of C-dots, Table S1. Fourier-transform infrared (FTIR) spectroscopy measurements suggested that Ce^4+^-ions are associated with the amine functionalities on the C-dots surface through the formation of metal-amine ligand bridges (Ce–N, 1041 cm^–1^),^70,71^Figure S2. X-ray photoelectron spectroscopy (XPS) measurements indicated that no CeO_2_ assemblies were formed, Figure S3.^72−74^ The sets of amino-modified DBA were covalently coupled to the free carboxylic acid functionalities associated with the Ce^4+^-ion-modified C-dots using 1-ethyl-3-(3-(dimethylamino)propyl) carbodiimide and *N*-hydroxysulfosuccinimide as coupling reagents. Figure 1A. The surface loading of the variable amino-functionalized DBA was calculated spectroscopically, see Figure S4 and the accompanying discussion. The loading of the different DBA on the C-dots in this study was very similar and corresponded to five aptamers per C-dot. Figure 1B depicts the oxidation of dopamine to aminochrome by H_2_O_2_ using the DBA-functionalized aptananozyme catalysts, Figure S5.

**Figure 2:**
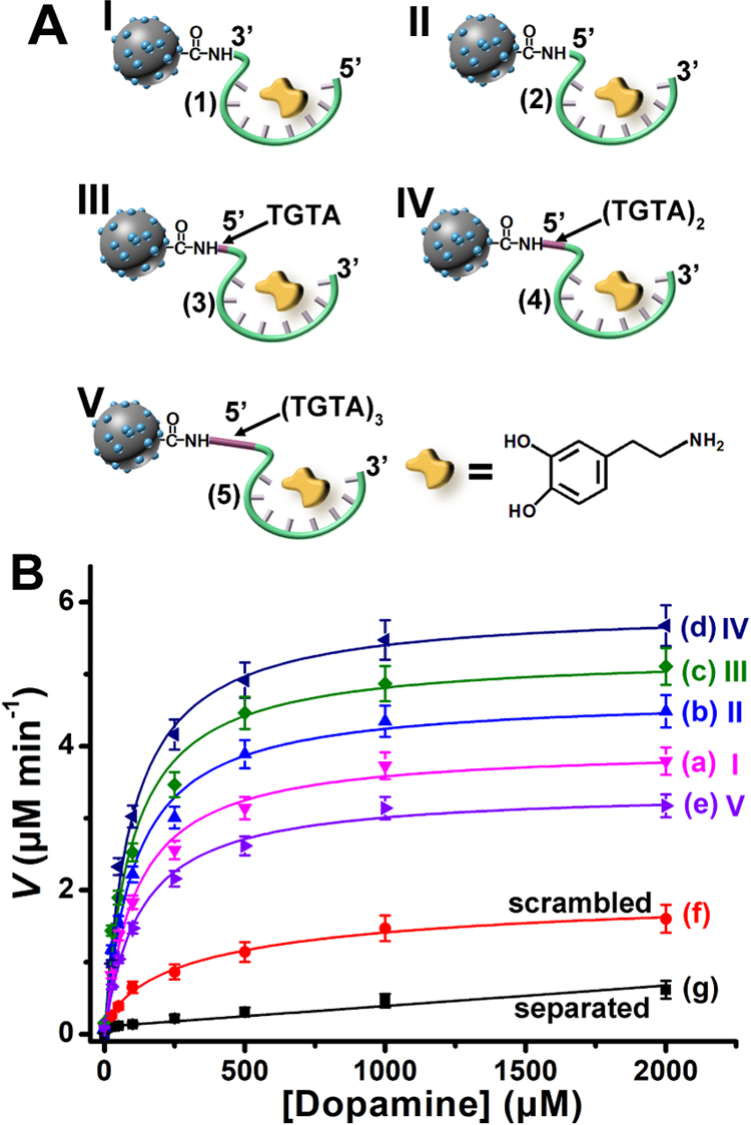
(A) Schematic configurations of the DBA-functionalized Ce^4+^-ion-modified C-dots, aptananozymes. (B) Rates of dopamine oxidation by H_2_O_2_ to aminochrome using variable concentrations of dopamine in the presence of (a) Aptananozyme I, (b) aptananozyme II, (c) aptananozyme III, (d) aptananozyme IV, (e) aptananozyme V, (f) the hybrid composed of scrambled DBA (**2a**) linked to Ce^4+^-ion-modified C-dots, and (g) the separated Ce^4+^-ion-modified C-dots and BDA (**2**).

Figure 2A depicts the configurations of the DBA-functionalized Ce^4+^-ion-modified C-dots, aptananozymes, that were examined as dopamine oxidation catalysts in the presence of H_2_O_2_. In aptananozyme I, the 3′-end-amino-modified aptamer (**1**) is linked to the Ce^4+^-ion-modified C-dots, whereas in configuration II, the 5′-end-modified aptamer (**2**) is linked to the Ce^4+^-ion-modified C-dots. In aptananozymes III, IV, and V, the 5′-end-amino-functionalized DBA aptamers are linked to Ce^4+^-ion-modified C-dots using one, two, and three (TGTA) spacer units of amino-functionalized DBA strands (**3–5**), respectively. In configuration V, the aptamer is linked to the Ce^4+^-ion-modified C-dots through a 12-base spacer unit. Figure 2B shows the rates of oxidation of dopamine to aminochrome by the different aptananozymes (I–V) in the presence of H_2_O_2_, 5 mM, using different concentrations of dopamine, curves (a–e). The kinetic curves corresponding to the time-dependent absorbance changes (at λ = 480 nm) of aminochrome formation were applied to derive the rates of oxidation of dopamine to aminochrome as a function of dopamine concentration by the respective aptananozymes (Figure S6, panels i–vi). For comparison, the rates of oxidation of dopamine to aminochrome by H_2_O_2_ in the presence of the separated Ce^4+^-ion-modified C-dots and the 5′-amino-modified DBA aptamer are presented, curve (g). All the aptananozymes reveal enhanced rates toward the oxidation of dopamine by H_2_O_2_, as compared to the separated constituents. The most efficient aptananozyme is aptananozyme IV, composed of the 5′-amino DBA aptamer linked to the C-dots by a 2×(TGTA) spacer bridge, and the catalytic activities of the aptananozymes I–IV follow the order I < II < III < IV. The aptananozyme V shows lowest catalytic activity toward oxidation of dopamine. The rate of dopamine oxidation by H_2_O_2_ to form aminochrome by the superior aptananozyme IV is *ca.* 14-fold enhanced as compared to the rate of oxidation of the dopamine substrate by the separated constituents. All the aptananozymes reveal saturation kinetics consistent with the saturation of the aptamer-binding sites by the dopamine substrate. The kinetic curves corresponding to the oxidation rates of dopamine as a function of the substrate (dopamine) concentration were analyzed following the Michaelis–Menten model, and the kinetic parameters characterizing the different aptananozymes are summarized in Table 1. The enhanced rates of dopamine oxidation by the aptananozymes, as compared to the oxidation of dopamine by the separated Ce^4+^-ion-modified C-dots and the aptamer constituents, are attributed to the binding of dopamine to the aptamer units and the concentration of the substrate at the catalytic sites “molarity effect”. In a further control experiment, an “aptananozyme” consisting of the scrambled sequence of the 5′-end-amino-modified DBA sequence (**2a**) was linked to the Ce^4+^-ion-modified C-dots. The rate of oxidation of dopamine by this aptananozyme II′ is depicted in Figure 2B, curve (f). The rate of oxidation of dopamine by the control aptananozyme II′ is substantially lower than the rate of oxidation of the substrate by the series of aptananozymes I–V, yet it is higher than the control system consisting of the separated Ce^4+^-ion-modified C-dots nanozyme and the 5′-end-amino-modified DBA aptamer (**2a**). The enhanced activity of the non-aptamer (scrambled) functionalized Ce^4+^-ion-modified C-dots is attributed to the electrostatic attraction of the positively charged dopamine to the negatively charged scrambled aptamer sequence (**2a**) associated with the particles. To understand the order of reactivities of aptananozymes toward the oxidation of dopamine, the dissociation constants of dopamine to the different aptananozymes were evaluated by isothermal titration calorimetry (ITC), and these are included in Table 1. For the series of aptananozymes revealing the order of reactivities I < II < III< IV, the catalytic performance of the aptananozymes follows the binding affinities of dopamine to the respective aptananozymes, Figure S7 and Table 1. As the binding affinity increases, the catalytic oxidations of the substrate are enhanced, which is consistent with the improved binding of the substrate to the aptamer sites. Interestingly, we find that the binding of the 5′-amino DBA aptamer to the C-dots yields a superior binding aptamer as compared to the 3′-amino DBA linked to the C-dots. Furthermore, we find that the introduction of the (TGTA) spacer groups improves the binding affinity of dopamine to the aptamer sites. Presumably, the Ce^4+^-ion-modified C-dots perturb the association of dopamine to the DBA aptamers, and the spacer bridges introduce the flexibility that facilitates the formation of the dopamine aptamer complexes. Indeed, as evident from Table 1, the *K*_d_ value of the DBA directly linked to the Ce^4+^-ion modified C-dots (aptananozyme II), *K*_d_ = 1.16 ± 0.06 μM is slightly higher than the *K*_d_ value of aptananozyme IV, where the aptamer is associated to the C-dots through 2×(TGTA) spacer unit, which is lower, *K*_d_ = 0.88 ± 0.04 μM, consistent with lower interfacial perturbation of the DBA aptamer binding properties. (Also, in general, the binding affinities of the DBA/Ce^4+^-ion modified C-dots conjugates toward dopamine are lower as compared to the binding affinity of dopamine to the free aptamer, *K*_d_ = 0.72 ± 0.03 μM). Interestingly, however, the aptananozyme V that reveals high-binding affinity toward dopamine, *K*_d_ = 0.91 ± 0.1 μM, shows the lowest catalytic activity among all aptananozymes I–V. Presumably, the long spacer bridges, composed of twelve bases, 3×(TGTA), separate spatially the dopamine-aptamer complex from the catalytic interface, resulting in lower catalytic performance. (For further evaluation of the ROS species involved with the aptananozymes catalyzed oxidation of dopamine by H_2_O_2_, vide infra).

**Table 1:** Kinetic Parameters Associated with the Aptananozyme-Catalyzed Oxidation of Dopamine by H_2_O_2_ to form Aminochrome^a^

aptananozyme	*V*_max_ (μM/min)	*K*_M_ (μM)	*k*_cat_ (10^–3^ s^–1^)	*K*_d_ (μM)
IV	5.92 ± 0.32	95 ± 9.6	1.49 ± 0.10	0.88 ± 0.04
III	5.28 ± 0.18	97 ± 13.5	1.33 ± 0.08	0.95 ± 0.03
II	4.69 ± 0.16	106 ± 14.1	1.18 ± 0.06	1.16 ± 0.06
I	3.98 ± 0.12	113 ± 13.4	1.00 ± 0.05	1.78 ± 0.07
V	3.39 ± 0.09	125 ± 12.5	0.85 ± 0.03	0.91 ± 0.10
II′ (scrambled)	1.8 ± 0.32	384 ± 56	0.45 ± 0.01	
separated DBA/Ce^4+^-C-dots	0.42		0.01	

a The *K*_d_ values corresponding to the binding of dopamine to the respective aptananozymes are also included.

The chiral properties of the aptamer binding sites suggested that chiroselective oxidation of chiral catechol substrates by H_2_O_2_ should proceed. Figure S8 demonstrates the aptananozyme II-stimulated chiroselective oxidation of l- or d-DOPA by H_2_O_2_ to yield l-/d-dopachrome. The oxidation of l-DOPA by H_2_O_2_ to form l-dopachrome is *ca.* twofold enhanced as compared to the oxidation of d-DOPA, Figure S8, curve (a) versus (b), and Table S2. These results are consistent with the higher binding affinity of l-DOPA to aptananozyme II, *K*_d_ = 2.6 ± 0.15 μM, as compared to the binding affinity of d-DOPA to aptananozyme II, *K*_d_ = 7.5 ± 0.13 μM, Figure S9. Figure S8, curves (c,d) show the rates of oxidation of l-/d-DOPA by H_2_O_2_ in the presence of the separated Ce^4+^-ion-modified C-dots and the diffusional aptamer (**2**). Besides the substantially lower oxidation rates of dopamine by the separated constituents, no noticeable chiroselective oxidation is observed. These results demonstrate the significance of the hybrid nanoparticles-aptamer aptananozyme structure in binding and concentrating l-/d-DOPA at the interface of catalytic nanoparticles for effective chiroselective oxidation of the chiral catechol substrates.

**Figure 3:**
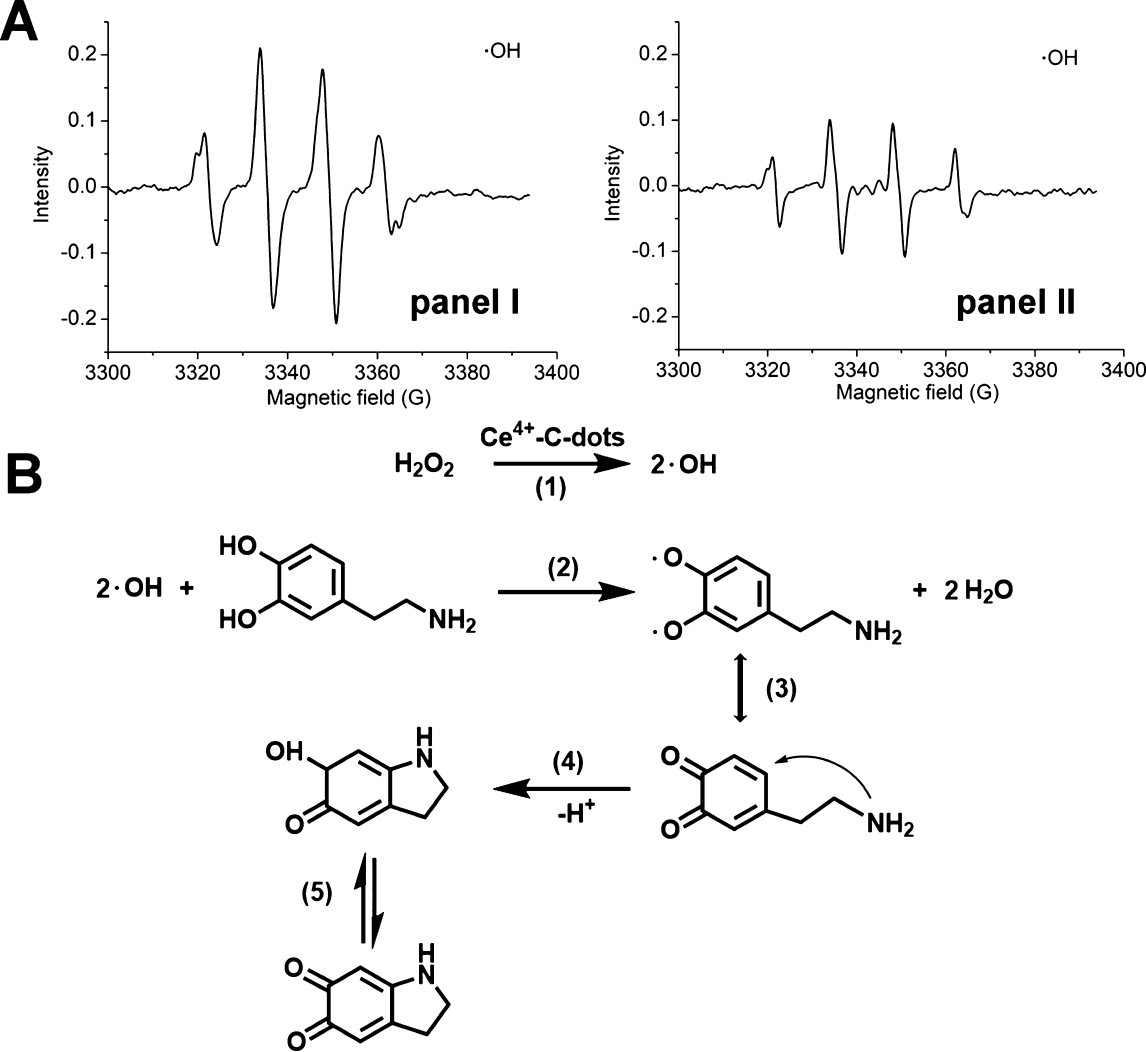
(A) EPR spectrum corresponding to the ^•^OH generated by aptananozyme IV in the presence of H_2_O_2_ (panel I) and in the presence of H_2_O_2_ and addition of dopamine (panel II). (B) The tentative mechanism leading to the oxidation of dopamine by the ^•^OH radicals.

Electron paramagnetic resonance (EPR) experiments were performed to identify the ROS participating in the aptananozyme-catalyzed oxidation of dopamine to aminochrome, Figure 3. We find that treatment of H_2_O_2_ with the aptamer (**4**)-functionalized Ce^4+^-ion-modified C-dots yields ^•^OH as the ROS product, Figure 3A, panel I. The addition of dopamine to the H_2_O_2_ solution treated with the (**4**)-aptamer-functionalized Ce^4+^-ion-modified C-dots results in the partial, yet significant, decrease of the ^•^OH band, consistent with the consumption of the radical, Figure 3A, panel II. Accordingly, the tentative mechanism leading to the oxidation of dopamine by ^•^OH was formulated, Figure 3B. It should be noted that the “bare” non-Ce^4+^-modified C-dots or the dopamine-aptamer modified C-dots lack any capacity to generate ^•^OH in the presence of H_2_O_2_ and lack any catalytic activity toward the oxidation of dopamine in the presence of H_2_O_2_ (*cf.*Figure S10).

**Figure 4:**
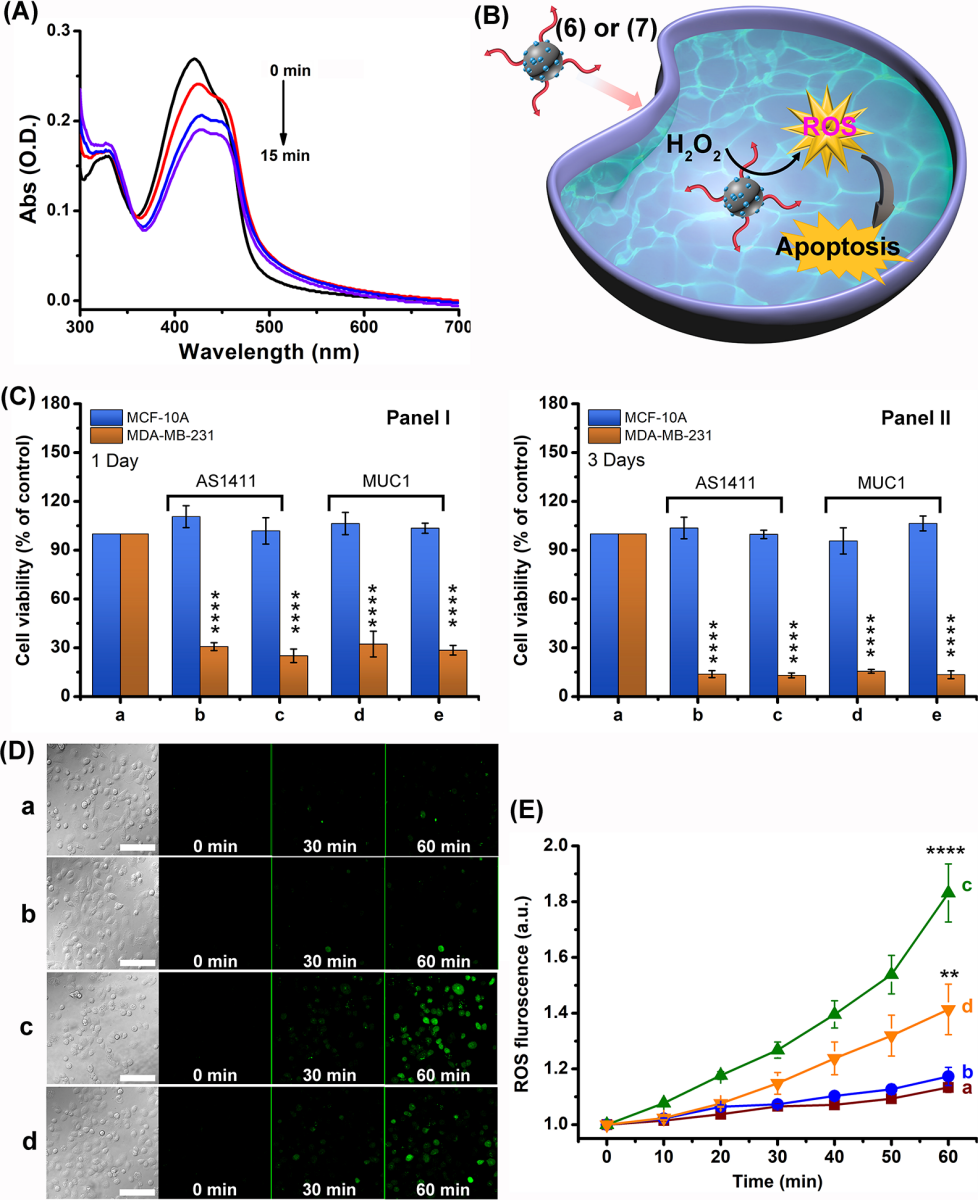
(A) Time-dependent absorbance spectra of 1,3-diphenylisobenzofuran, DPBF, upon reaction with the ROS species generated by AS1411-functionalized Ce^4+^-ion-modified C-dots in the presence of H_2_O_2_. (B) Schematic chemodynamic treatment of cancer cells targeted by aptamer-functionalized Ce^4+^-ion-modified C-dots catalyzing the H_2_O_2_-mediated generation of ROS. (C) Cytotoxicity of the aptamer-functionalized Ce^4+^-ion-modified C-dots toward MDA-MB-231 breast cancer cells and control MCF-10A epithelial breast cells. Panel I—after treatment for 1 day with AS1411 aptamer-functionalized Ce^4+^-ion-modified C-dots at (b) 2.5 and (c) 5.0 μg mL^–1^ and with (MUC1-aptamer-functionalized Ce^4+^-ion-modified C-dots at (d) 2.5 and (e) 5.0 μg mL^–1^. Panel II—after treatment for 3 days with AS1411 aptamer-functionalized Ce^4+^-ion-modified C-dots at (b) 2.5 and (c) 5.0 μg mL^–1^ and with MUC1-aptamer-functionalized Ce^4+^-ion-modified C-dots, at (d) 2.5 and (e) 5.0 μg mL^–1^. Columns (a) correspond to non-treated cells. (D) Confocal bright-field and fluorescence images corresponding to the time-dependent formation of ROS intermediates probed by the C-DCDHF-DA dye in MDA-MB-231 cancer cells: (a) non-treated cells, (b) Ce^4+^-ion-modified C-dots functionalized with a scrambled AS1411 aptamer sequence, (c) AS1411 aptamer-functionalized Ce^4+^-ion-modified C-dots, (d) MUC1 aptamer-functionalized Ce^4+^-ion-modified C-dots. Scale bar corresponding to 100 μm (E) Integrated time-dependent fluorescence intensities at λ_em_ = 517 nm of *N* = 3 experiments shown in (D). Significant results were evaluated using the *T*-test; **P < 0.01, ****P < 0.0001.

Hydrogen peroxide is present in cancer cells or inflamed tissues.^61−64^ Indeed, recent research efforts have demonstrated that ROS intermediates generated by nanozymes can act as antibacterial agents and as active toxic agents against cancer cells.^75,76^ In fact, the formation of ROS species by photodynamic therapeutic treatment of cancer cells demonstrated effective cytotoxicity toward cancer cells, and recent research efforts suggested the application of nanozyme-generated ROS species as a means for chemodynamic treatment of cancer cells.^77,78^ The drawbacks of this concept rest, however, on the limited cellular permeability and lack of selectivity of the nanoparticles affecting cancer as compared to normal cells. The effective permeation of C-dots into cells,^79,80^ suggests that functionalization of Ce^4+^-ion-modified C-dots with cell-specific aptamers could be an ideal method to develop effective aptananozymes for chemodynamic treatment of cancer cells. That is, the functionalization of the Ce^4+^-ion-modified C-dots with cancer cell-specific aptamers would enhance the targeted permeation of the nanoparticles into cancer cells, and the H_2_O_2_ presented in the cancer cells will act as the aptananozyme substrate for the selective generation of ^•^OH as a cytotoxic agent in the cancer cells/tissues. Accordingly, the Ce^4+^-ion-modified C-dots were functionalized with the amino-modified AS1411 aptamer, (**11**), that binds to the nucleolin receptor associated with various cancer cells,^81,82^ or with the MUC1 aptamer (**12**) that binds to different cancer cells, for example, breast cancer cells.^66,83^ In vitro experiments, the formation of ^•^OH in the presence of H_2_O_2_ and the AS1411-functionalized Ce^4+^-ion-modified C-dots in the presence of H_2_O_2_. Figure 4A shows the absorbance changes of the ROS probe 1,3-diphenylisobenzofuran, DPBF, upon reacting H_2_O_2_ with AS1411-functionalized Ce^4+^-ion-modified C-dots in the presence of the DPBF probe. The time-dependent depletion of the absorbance spectra of DPBF is consistent with the formation of ROS products.^84,85^ Control experiments revealed that the AS1411-functionalized Ce^4+^-ion-modified C-dots in the absence of H_2_O_2_ or the application of H_2_O_2_ on the probe agent in the absence of the AS1411-functionalized Ce^4+^-ion-modified C-dots had very little effect on the ROS probing label, Figure S11. These experiments confirm the in vitro generation of the ROS product (^•^OH) by the AS1411-functionalized Ce^4+^-ion-modified C-dots. It should be noted that the non-Ce^4+^-modified C-dots functionalized with the AS1411 aptamer or the MUC1 aptamer did not show any ROS product generation capacity in the presence of H_2_O_2_.

Subsequently, the chemodynamic cytotoxic treatment of cancer cells with the AS1411- or the MUC1-functionalized Ce^4+^-ion-modified C-dots generating the ROS agents was then examined, Figure 4B. (For the stability of the aptamer functionalized Ce^4+^-ion-modified C-dots in cellular media, see Figure S12 and accompanying discussions). Figure 4C shows the viability of MDA-MB-231 breast cancer cells and MCF-10A epithelial breast cells treated with different amounts of the AS1411 aptamer-functionalized Ce^4+^-ion-modified C-dots (2.5 and 5.0 μg mL^–1^) for a time interval of 1 day and with different amounts of the MUC1 aptamer-functionalized C-dots (2.5 and 5.0 μg mL^–1^) for a time interval of one day, panel I. Similarly, the viability of these cells was subjected to different amounts of AS1411 aptamer-modified Ce^4+^-ion-C-dots and of the MUC-1 aptamer-functionalized Ce^4+^-ion-C-dots for a time interval of 3 days, panel II. The results demonstrate that no noticeable cell death of the MCF-10A normal breast cells is detected upon treatment with the AS1411 aptamer-modified Ce^4+^-ion-C-dots or the MUC1 aptamer-functionalized Ce^4+^-ion-C-dots for 1 or 3 days, and effective chemodynamic cytotoxicity toward the MDA-MB-231 breast cancer cells is observed. Treatment of the MDA-MB-231 breast cancer cells with the AS1411 aptamer-functionalized Ce^4+^-ion-modified C-dots for 1 and 3 days leads to *ca.* 75 and 85% cell death, respectively, whereas subjecting the MUC1 aptamer-functionalized Ce^4+^-ion-C-dots to the MDA-MB-231 breast cancer cells shows after 1 and 3 days of cell death corresponding to *ca.* 70 and 85%, respectively. Beyond the high cytotoxicity of aptamer-functionalized Ce^4+^-ion-C-dots, the selective cytotoxicity toward the cancer cells is noteworthy. The resulting selectivity is attributed to the aptamer-guided permeation of the Ce^4+^-ion-modified C-dots into the cancer cells by nucleolin on AS1411 and MUC1 receptors associated with the cancer cells. Indeed, Figure 4D shows the temporal confocal fluorescence images of the MDA-MB-231 cancer cells stained with the di(acetoxymethyl ester)-6-carboxy-2′,7′-dichlorodihydrofluorescein diacetate (C-DCDHF-DA), a ROS detection dye (λ_ex_ = 488 nm; λ_em_ = 517 nm), upon non-treatment of sample, and upon treatment with the scrambled AS1411 aptamer-functionalized Ce^4+^-ion-modified C-dots, panels a and b, respectively. No fluorescence signals are detected, which is consistent with the lack of permeation and generation of ROS by the particles in these cells. In turn, Figure 4D, panels c and d, show the temporal confocal fluorescence microscopy images of the C-DCDHF-DA-stained MDA-MB-231 cancer cells treated with the AS1411 aptamer-functionalized Ce^4+^-ion-modified C-dots and with the MUC1 aptamer-functionalized Ce^4+^-ion-modified C-dots. Effective time-dependent increase of the green fluorescence in the cells is observed, consistent with the build-up of ROS intermediates in the cells. (For further temporal confocal fluorescence images of the MCF-10A epithelial breast cells, see Figure S13). Figure 4E depicts the integrated time-dependent fluorescence changes of the stained cancer cells and control systems recorded from four different frames of cancer cells upon treatment with the AS1411 aptamer or the MUC-1 aptamer-functionalized Ce^4+^-ion-C-dots. The temporal integrated fluorescence intensities reflect the time-dependent accumulation of the ROS intermediates in the respective cells. The results demonstrate selective and effective formation of ROS in the AS1411 aptamer- or in the MUC1 aptamer-functionalized Ce^4+^-ion-modified C-dots, consistent with the selective chemodynamic cytotoxicity of the particles toward the cancer cells. It should be noted that the non-Ce^4+^-modified C-dots functionalized with the AS1411 aptamer or the MUC1 aptamer did not show any intracellular formation of ROS products in the MDA-MB-231 cancer cells.

**Figure 5:**
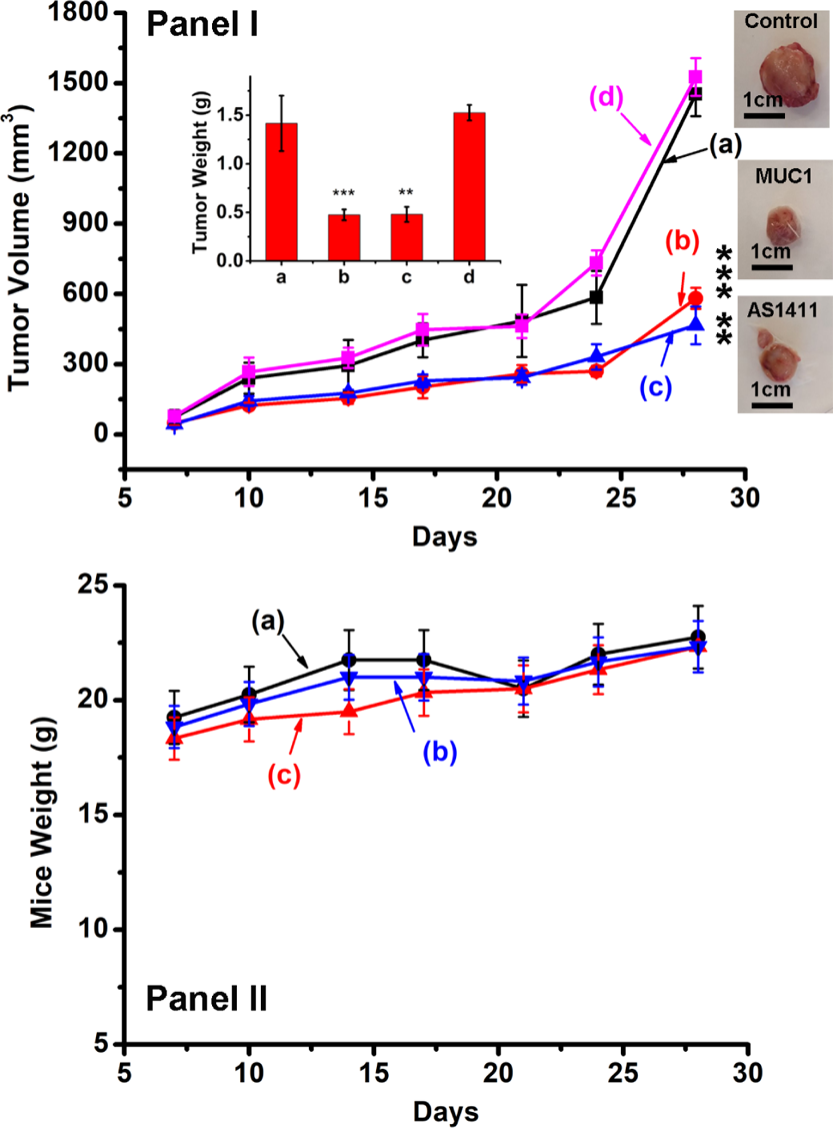
Panel I—Xenograft tumor volume profiles; inset—final tumor weight extracted from mice. Panel II—Corresponding body weight changes of xenograft epithelial MDA-MB-231 breast cancer tumors bearing NOD-SCID mice that were treated with (a) Ce^4+^-ion-modified C-dots, (b) AS1411 aptamer-functionalized Ce^4+^-ion-modified C-dots, (c) MUC1 aptamer-functionalized Ce^4+^-ion-modified C-dots, and (d) PBS buffer. All results were presented as mean ± SEM. Significant results were evaluated using the *T*-test; **P < 0.01, ***P < 0.001.

Preliminary in vivo experiments that follow the chemodynamic treatment of MDA-MB-231 breast cancer tumors by the aptamer-functionalized Ce^4+^-ion-modified C-dots were performed. In these experiments, xenograft MDA-MB-231 breast xenograft cancer tumors bearing NOD-SCID mice were subjected to the intra-tumor injection of the AS1411 aptamer- and MUC1 aptamer-functionalized Ce^4+^-ion-modified C-dots. Figure 5, panel I, depicts the average time-dependent volume changes of the tumors in different mice samples treated along 28 days with different C-dots. While the mice treated with the bare Ce^4+^-ion-modified C-dots revealed after 28 days an average size of *ca.* 1400 mm^3^, the tumors treated with the AS1411 aptamer-functionalized Ce^4+^-ion-modified C-dots or MUC1 aptamer-functionalized Ce^4+^-ion-modified C-dots revealed after this time interval a substantially lower volume corresponding to *ca.* 400 mm^3^. The inhibited growth of the tumors treated with the aptamer-functionalized Ce^4+^-ion-modified C-dots particles into the cells that result in effective intracellular cytotoxic ROS intermediates generated by the aptananozyme in the presence of H_2_O_2_. Figure 5, panel II, shows the average weight of the mice treated with the different C-dots. No loss in the weight of the mice with the C-dots is observed, indicating that all C-dots are non-toxic toward the mice. To further understand the enhanced chemodynamic cytotoxicity of the AS1411 aptamer-functionalized Ce^4+^-C-dots as compared to the MUC1 aptamer-modified Ce^4+^-C-dots toward the MDA-MB-231 cells or tumors, we examined the interactions (binding efficacies) of the two kinds of aptamer-modified C-dots with the MDA-MB-231 cells. While we do not know the concentrations of the nucleolin or MUC1 receptors associated with cells, and pure quantities of the receptors are not available, we probed the binding affinities of the two aptamer-modified Ce^4+^-ion-modified C-dots with a constant concentration of MDA-MB-231 cells, 200,000 cells mL^–1^, using ITC measurements. We find that the dissociation constraints, *K*_d_, of the AS1411 aptamer-functionalized Ce^4+^-ion-modified C-dots and of the MUC1 aptamer-modified Ce^4+^-ion-modified C-dots to the cells correspond to 4.3 and 7.2 μM, respectively. The lower *K*_d_ value of the AS1411 aptamer-modified C-dots points to a higher binding affinity of these nanoparticles to the MDA-MB-231 cancer cells. This is consistent with the chemodynamic activity of the AS1411 aptamer-functionalized Ce^4+^-ion-modified C-dots as compared to the MUC1 aptamer-functionalized Ce^4+^-ion-modified C-dots. (Enhanced cell permeation and enhanced ROS generation efficacies in the cells and tumors). (For further histological analysis of MDA-MB-231 tumor treated with the Ce^4+^-ion-functionalized C-dot modified with the AS1411 aptamer and the MUC1 aptamer, see Figure S14 and the accompanying discussion.)

## Conclusions

The present study introduced Ce^4+^-ion-modified C-dots functionalized with the DBA as superior aptananozymes for the oxidation of dopamine by H_2_O_2_ to aminochrome. Structure–catalytic function relationships in a set of aptananozymes catalyzing the reaction, the chiroselective oxidation of d/l-DOPA, and the participation of ROS intermediates in the reactions were demonstrated. The conjugation of other aptamers, for example, anti-pesticide aptamers to the peroxidase mimicking Ce^4+^-ion-modified C-dots could yield aptananozyme catalyzing other chemical transformations, particularly environmentally hazardous wastes. Alternatively, the integration of other nanoparticle/nanocluster nanozymes with the Ce^4+^-ion-modified C-dots aptananozyme systems, could yield hybrid nanozyme bioreactors aptananozyme systems driving effective biocatalytic cascades. In addition to the integration of aptamers with the peroxidase-mimicking, ROS-generating, Ce^4+^-ion-modified C-dots yielded effective and selective functional aptananozymes for biomedical applications. The aptamer conjugates targeted and facilitated the permeation of the ROS-generating nanoparticles into cancer cells. This was exemplified with the functionalization of the Ce^4+^-ion-modified C-dots with the AS1411 aptamer and the MUC1 aptamer for the chemodynamic treatment of MDA-MB-231 breast cancer cells and the in vivo inhibition of MDA-MB-231 tumor growth in mice. The results suggest that modification of the Ce^4+^-ion-modified C-dots with other target-specific aptamers could lead to other medical applications of the aptananozymes. Furthermore, recent studies on introduced NMOFs as effective anti-cancer drug carriers^86,87^ and demonstrated the catalytic activities of different NMOFs to yield ROS agents.^25^ Thus, by loading such catalytic NMOFs with anti-cancer drugs and their functionalization with targeting aptamer units, superior therapeutic carriers revealing cooperative chemodynamic and chemotherapeutic functionalities may be anticipated.

## Experimental Section

Sequences used in the study:

(**1**) Amino-DBA for 5′-linked aptananozyme (I): 5′-NH_2_-CGACGCCAGTTTGAAGGTTCGTTCGCAGGTGTGGAGTGACGTCG-3′.

(**1a**) Amino-scrambled DBA for 5′-linked aptananozyme: 5′-NH_2_-GACTAGCGTGTGTGATGGGACCTTAGGCCGTCACGGGGCTTAGT-3′.

(**2**) Amino-DBA for 3′-linked aptananozyme (II): CGACGCCAGTTTGAAGGTTCGTTCGCAGGTGTGGAGTGACGTCG-NH_2_-3′

(**3**) Amino-DBA with (TGTA) spacer for 5′-linked aptananozyme (III): 5′-NH_2_-TGTA-CGACGCCAGTTTGAAGGTTCGTTCGCAGGTGTGGAGTGACGTCG-3′

(**4**) Amino-DBA with (TGTA)_2_ spacer for 5′-linked aptananozyme (IV): 5′-NH_2_-TGTATGTA-CGACGCCAGTTTGAAGGTTCGTTCGCAGGTGTGGAGTGACGTCG-3′

(**5**) Amino-DBA with (TGTA)_3_ spacer for 5′-linked aptananozyme (V): 5′-NH_2_-TGTATGTATGTA-CGACGCCAGTTTGAAGGTTCGTTCGCAGGTGTGGAGTGACGTCG-3′

(**6**) Amino-AS1411: 5′-NH_2_-AAGGTGGTGGTGGTTGTGGTGGTGGTGGTTT-3′

(**7**) Amino-AS1411: 5′-NH_2_-AAGCAGTTGATCCTTTGGATACCCTGG-3′

For the synthesis of C-dots, the detailed conjugation of the aptamers to the C-dots, the characterization of the aptamer and C-dots conjugates and the kinetic measurements see Supporting Information.

## Supporting Information Available

Experimental section, quantification of the aptamer and Ce^4+^-ions-modified C-dots, TEM image, XPS and FTIR spectra, time-dependent absorbance changes upon the oxidation of different concentrations of dopamine, ITC measurements, in vitro and in vivo experiments, and histopathological analysis (PDF)
