# Supplementary material for: Aptamer-Functionalized Ce4+-Ion-Modified C-Dots: Peroxidase Mimicking Aptananozymes for the Oxidation of Dopamine and Cytotoxic Effects toward Cancer Cells
Source: ACS Appl Mater Interfaces. 2022 Dec 7;14(50):55365–75. doi: 10.1021/acsami.2c16199 (PMC9782376; doi:10.1021/acsami.2c16199)
Supplement: Supplementary file 1 — am2c16199_si_001.pdf [file am2c16199_si_001.pdf]

Supporting Information

Aptamer-Functionalized Ce^{4+}-ion-Modified C-dots: Peroxidase Mimicking aptananozymes for the oxidation of Dopamine and Cytotoxic Effects Toward Cancer Cells

Yu Ouyang[1,§] Michael Fadeev[1,§] Pu Zhang[1], Raanan Carmieli[2], Yang Sung Sohn[3], Ola Karmi[3], Yunlong Qin[1], Xinghua Chen[1], Rachel Nechushtai[3], Itamar Willner[1*]

1. The Institute of Chemistry, The Hebrew University of Jerusalem, Jerusalem 91904, Israel.

2. Department of Chemical Research Support, Weizmann Institute of Science, Rehovot, 76100, Israel.

3. Institute of Life Science， The Hebrew University of Jerusalem, Jerusalem 91904, Israel.

E-mail: Itamar.willner@mail.huji.ac.il

[§] Yu Ouyang and Michael Fadeev contributed equally to this work.

Instruments. Absorption spectra were recorded on a UV-2450 spectrophotometer (UV, Shimadzu). Kinetic measurements were performed at 25 °C using a Biotek Synergy H1 microplate reader, equipped with a Biotek dual dispensing unit, and using Corning 3696 96-hald-well plates. Dissociation constants were evaluated using Isothermal titration calorimetry (ITC) instrument (Malvern instruments MicroCal PEAQ-ITC). X-ray photoelectron spectroscopy (XPS) measurements were performed with an Axis Ultra photoelectron spectrometer from Kratos Analytical. Fourier-transform infrared spectroscopy (FTIR) measurements were performed using a Nicolet iS50 FTIR Spectrometer. Transmission Electron Microscope was performed on a Tecnai G2 Spirit TWIN T12. Ce^{4+}-ions-modified C-dots were characterized by coupled plasma mass spectrometry using an Inductively Coupled Plasma-Mass Spectrometry (ICP) instrument. Electron paramagnitic resonance (EPR) measurements were carried out at room temperature using a Bruker ELEXYS E500 spectrometer operating at X-band frequencies (9.5 GHz) and a Bruker ER4102ST resonator.

Preparation of C-dots and their modification with Ce^{4+}-ions. The C-dots were synthesized according to Wang *et al*[1]. Citric acid and urea were mixed in water and heated for 4-5 min in a 750 W microwave. This solid was then transferred to a vacuum oven and heated at 60℃ for 1 h to remove the residual small molecules. An aqueous solution of the C-dots was purified in a centrifuge (3000 g, 20 min) to remove large or agglomerated particles. The resulting colored (brown) aqueous solution remained indefinitely stable at various concentrations. The obtained C-dots solution was purified by 3 kDa and 10 kDa cutoff filters under 9168 g for 30 min, respectively. Finally, the

C-dots solution of below 3 kDa was frozen by liquid nitrogen and dried through lyophilization. The Ce^{4+}-ion-modified C-dots were prepared as follows: ammonium cerium (IV) nitrate (0.2 M, 50 μL) and C-dots (10 mg mL^{-1}, 500 μL) were mixed and shaking for 30 min. The mixture was under centrifugation at 18000 g for 5 min to collect the sediment. The wash process is repeated for several times.

Modification of Ce^{4+}-ion-modified C-dots with amino-modified aptamers or amino-functionalized control strands.

A 50 uL solution of Ce^{4+}-ion-modified C-dots (4 mg mL^{-1}) were subsequently added to equal volumes of 20 μL EDC (50 mM) and 50 μL sulfo-NHS (50 mM) in MES buffer (5 mM, pH 5.5) and left to react for 15 min. The above mixture was sequentially added 100 μL of 100 mM phosphate buffer, pH 7.2 and 100 μL of amino-functionalized aptamer (20 μM in 100 mM phosphate buffer, pH 7.2). The coupling reaction was performed at room temperature for 12 h. The solution after the reaction was purified by 3 kDa cutoff filters under 10000 g for 30 min and repeat for 4 times.

Evaluation of the loading of the aptamers on Ce^{4+}-ion-modified C-dots. After loading the amino-functionalized aptamers to the Ce^{4+}-ion-modified C-dots, the concentration of the Ce^{4+}-ions was determined by the ICP. Following the same procedure of preparation of attaching the aptamers to Ce^{4+}-ion-modified C-dots, the ratios of C-dots to Ce^{4+}-ions are very similar to each other (1 : 0.3, mg mL^{-1}).

The ratios between the Ce^{4+}-ion-modified C-dots and aptamer could be determined by the relative calibration curves. As we can see from the Figure S4A and S4B, the two calibration curves at 260 and 338 nm were formed by the different concentrations of

the Ce^{4+}-modified C-dots solution by UV spectra. Figure S4C show the UV spectra of 5′-DBA associated with Ce^{4+}-ion-modified C-dots. After we attach the NH_2-aptamers to the C-dots, the calibration curve at 330 nm is required to quantify the amount of C-dots, while the absorbance of C-dots at 260 nm is obtained. By the subtraction of the spectrum at 260 nm, the net absorbance of the nucleic acids associated with the C-dots is evaluated. It should be noted that multiple groups of experiments were executed to achieve the similar ratio of aptamers to Ce^{4+}-ion-modified C-dots.

Kinetic measurements with aptananozymes. Kinetic measurements were performed at 25 °C using a Biotek Synergy H1 microplate reader equipped with a Biotek dual dispensing unit and using Corning 3696 96-well plates. For dopamine oxidation, the aptananozymes (0.8 μg mL^{-1}) were dissolved in 5 mM MES buffer, pH 5.5, 5 mM $MgCl_2$, 100 mM NaCl, and 10 μL of dopamine, consisting of variable concentrations which were added to the respective wells. The slightly acidic conditions were selected to prevent background aerobic oxidation of dopamine. Subsequently, 10 μL of H_2O_2 (final concentration 1 mM) was dispensed into each well, and the absorbance values of the oxidized products (absorbance at 480 nm, ε = 3058 M^{-1} cm^{-1}) were measured in the different wells for a time interval of 30 minAs for the L-DOPA and D-DOPA oxidation process, the aptananozyme I (0.8 ug mL^{-1}) was dissolved in in 50 mM phosphate buffer solution (pH 7.2, containing 100 mM NaCl and 5 mM $MgCl_2$), and 10 μL of L/D-DOPA, consisting of variable concentrations which were added to the respective wells. Subsequently, 10 μL of H_2O_2 (final concentration 1 mM) was dispensed into each well, and the absorbance values of oxidized products (absorbance at 475 nm, ε = 3600 M^{-1}

cm^{-1}) were measured in the different wells for a time interval of 30 min.

EPR measurements. Radical species such as $^{\cdot}$OH, were detected using the EPR spin trapping technique coupled with a spin trap 3,4-dihydro-2-methyl-1,1-dimethylethyl ester-2H-pyrrole-2-carboxylic acid-1-oxide (BMPO). Typically, five mixtures of H_2O_2 (5 mM) and aptananozyme I (10 μg mL^{-1}), H_2O_2 (5 mM) including BMPO (0.01 M). The EPR measurements were taken with the following conditions: Centerfield, 3352.00 G; Sweepwidth, 100.0 G; MW Power, 20.02 mW; Modulation Amplitude, 1.00 G; Modulation Frequency, 100.00 kHz; No of points 512; Conversion Time, 40.96 ms; Conversion Time, 40.96 ms.

ITC evaluation of the K_d values of dopamine or D-/L-DOPA bound to DBA-functionalized aptananozymes. A stock solution of the respective aptananozyme, 20 μM, in a 5 mM MES buffer pH 5.5, 100 mM NaCl and 5 mM $MgCl_2$ for the DBA-aptananozymes were prepared. Stock solutions of dopamine (600 μM), L-DOPA (1 mM), D-DOPA (1 mM) in their respective buffers were prepared. The respective aptananozymes loaded into the sample cell, 280 μL, and the ITC instrument syringe was with the respective ligand stock solution. The aptananozyme sample was titrated by injecting repeated 2 μL aliquots of the respective ligand into the measurement cell (total 15-18 injections). The heat difference between the measuring and reference cell, upon each injection were evaluated under the following conditions: Cell reference power set at 41.9 μW, syringe rotation rate set to 750 RPM, initial delay of 60 seconds before the first injection to ensure equilibration of sample cell, injections spaced at 150-300 seconds to ensure baseline stabilization between measurements. The C-value (C-

value = [aptamer in cell]/K_d of free aptamer) were evaluated before the experiments and were in the range of 3-20 for all aptananozymes. Each binding experiment was repeated $n = 2$. The resulting K_d fitting was performed using the instrument (MicroCal PEAQ-ITC Analysis Software) with the "one set of sites" binding model and free parameters for K_d, Hill coefficient, and ΔH. All fitted K_d curves yielded a Hill coefficient of 0.9 to ~1, implying a 1:1 complex between the ligands and the aptamers.

Cell experiments *Cell culture:* Normal breast cells (MCF-10A) were maintained in complete growth medium consisting of 1:1 mixture of Dulbecco's modified Eagle's medium and Ham's F12 medium supplemented with horse serum (5%), epidermal growth factor (20 ng mg^{-1}), cholera toxin (CT, 0.1 μg mL^{-1}), insulin (10 μg mL^{-1}), hydrocortisone (500 ng mL^{-1}), and penicillin/streptomycin (1 unit mL^{-1}). Human breast cancer cells (MDA-MB-231) were grown in RPMI-1640 medium supplemented with 10% FCS, L-glutamine, and antibiotics (Biological Industries). Cells were plated one day prior to the experiment on 96-well plates for cell viability.

Cell viability experiments: Cell viability was assayed after incubation of Ce^{4+}-ion-modified C-dots, AS1411-Ce^{4+}-ion-modified C-dots, MUC-1-Ce^{4+}-ion-modified C-dots in MCF-10A, MDA-MB-231 cells planted at a density of 1.2×10^4 cells per well in 96-well plates. After 6 hours incubation with the C-dot- Ce^{4+} NPs, cells were washed intensively with growth medium. After washing, cells were replenished with full medium and further incubated for 3 days. The cell viability was determined after 1 day and 3 days with the fluorescent redox probe, Presto-Blue. The fluorescence of Presto-

Blue was recorded on a plate-reader (Tecan Safire) after 1 h of incubation at 37 °C (λ_{ex} = 560 nm; λ_{em} = 590 nm).

ROS production measurement: ROS production in cancer cells compared to normal cells was determined by incubating cells with Ce^{4+}-ion-modified C-dots, AS1411-Ce^{4+}-ion-modified C-dots, MUC-1-Ce^{4+}-ion-modified C-dots for 3 h and then by incubating cells containing C-dot-Ce^{4+}, C-dot- Ce^{4+}-AS1411, C-dot- Ce^{4+}-MUC-1at 37°C with 10 μM of Carboxy-H_2DCFDA-AM in HEPES-buffered saline (HBS) supplemented with 10 mM glucose. This nonfluorescent molecule is readily converted to a green-fluorescent form when the acetate groups are removed by intracellular esterase and oxidation by the activity of ROS within the cell. The conversion of the non-fluorescent probe to the green-fluorescent form was measured for 1 h at 37°C under the confocal microscopy (the Olympus FV3000 confocal laser-scanning microscope) (λ_{ex} = 488 nm; λ_{em} = 517 nm) and all images were analyzed with image J.

Mice experiments. Female NOD-SCID mice were used for detecting breast cancer xenograft tumor cell progression of MDA-MB-231 cells which were injected subcutaneously. The experiment was approved by the Authority for Biological and Biomedical Models at the Hebrew University, ethical number is NS-21-16745-4. MDA-MB-231 of 6X106 cells/mouse were injected subcutaneously to the flack of each mouse. Tumor mass was generated after 7 days in a volume that is around 80-100 mm^3, then the injections of the treatment were done intra-tumoral (IT) (2 times/week) in total 7 injections, by using C-dot-Ce^{4+} for the control group, compared to MUC-1 modified

C-dot-Ce^{4+} or AS1411-modified C-dot-Ce^{4+}, all particles were injected in a volume of 100 μL of the amount of 50μg/mouse. NPs were prepared in a final concentration of 1 mg mL^{-1}. For each group we used 4 mice. Tumor was majored every 3 days before the following injection to evaluate the width and the height, then tumor volume (mm3) was measured using the equation of (Width2XHigeht)/2. Toxicity of the treatment was evaluated by the mice weight change (g) that was measured once a week. All results were presented as mean ± SEM.

The histochemistry and immunohistochemistry methods included the following protocols: Histochemistry and immunohistochemistry were performed on paraffin-embedded tissue samples as described previously (Landolina, Nadine, et al. "Activation of Siglec-7 results in inhibition of in vitro and in vivo growth of human mast cell leukemia cells." Pharmacological Research 158 (2020): 104682.) Then they were cut into 4-μm sections and prepared as described in (Landolina, Nadine-----). Slides were stained with Hematoxylin and Eosin (H&E) and histological analysis was performed using standard procedures (Shalom, Batel, et al. "Vav1 accelerates Ras-driven lung cancer and modulates its tumor microenvironment." Cellular Signalling 97 (2022): 110395.). Immunohistochemical against Caspase 3 antibody staining were performed as described (Landolina, Nadine, et al. "Activation of Siglec-7 results in inhibition of in vitro and in vivo growth of human mast cell leukemia cells." Pharmacological Research 158 (2020): 104682.)

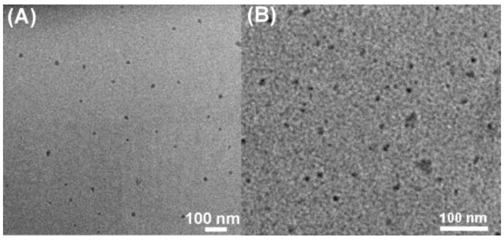

Figure S1. (A) TEM image of C-dots. (B) TEM image of aptananozymes.

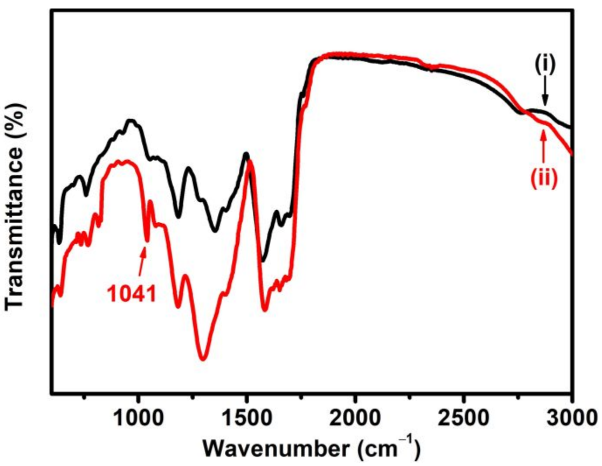

Figure S2. FTIR spectra of (i) C-dots, and (ii) Ce^{4+}-ions-modified C-dots.

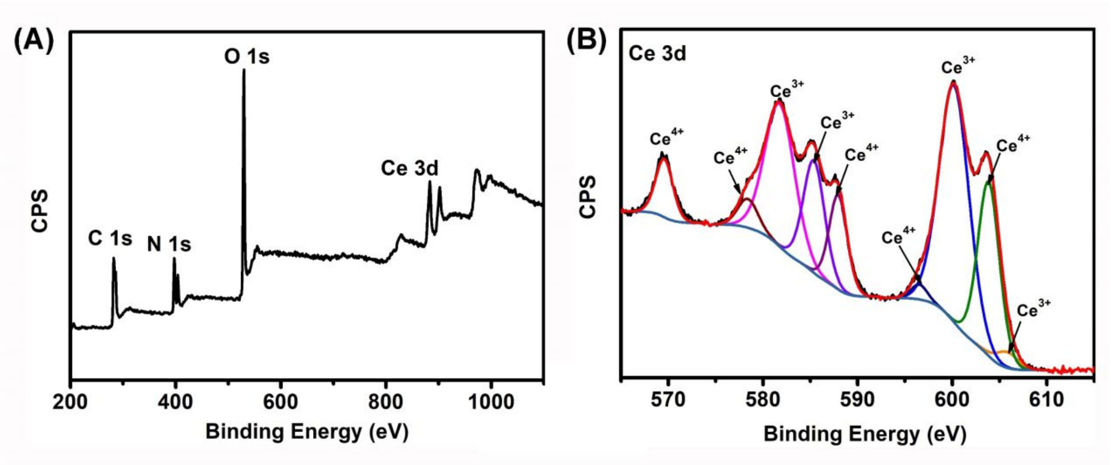

Figure S3. (A) Survey XPS spectrum of Ce^{4+}-ion-modified C-dots. (B) High-resolution XPS spectrum of Ce (3d) in Ce^{4+}-ion-modified C-dots.

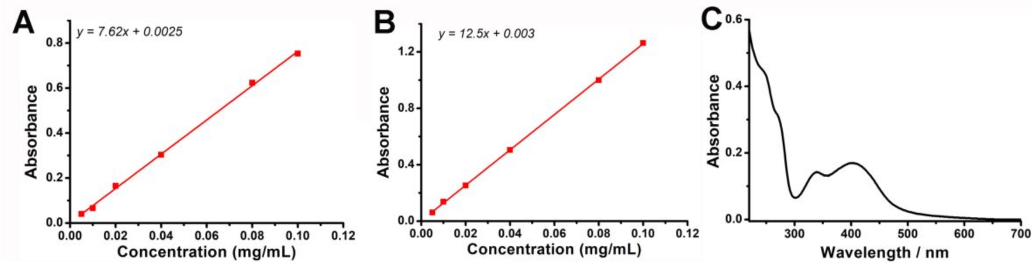

Figure S4. The calibration curves of C-dots-Ce^{4+} at 338 nm (A) and 260 nm (B), respectively. (C) The UV curve of 5′-DBA associated with Ce^{4+}-modified C-dots (aptananozyme I). (The way to evaluate aptamers on the C-dots see part of "Evaluation of the loading of the aptamers on Ce^{4+}-ion-modified C-dots.").

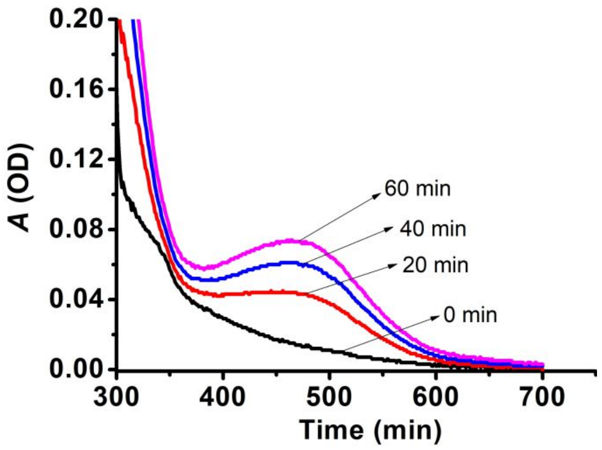

Figure S5. Time-dependent absorption spectra of oxidized dopamine in the presence of 0.2 μg mL^{-1}Aptananozyme I, 1 mM dopamine, 5 mM H_2O_2.

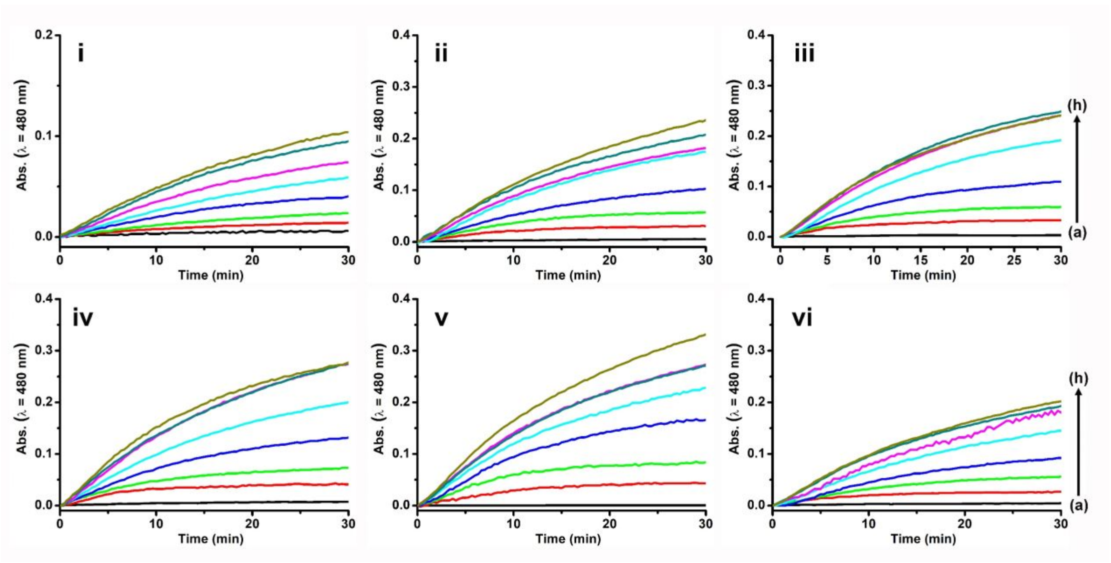

Figure S6. Time-dependent absorbance changes (λ = 480 nm) upon the oxidation of different concentrations of dopamine to aminochrome by the aptananozymes I-V: (a) 0, (b) 25, (c) 50, (d) 100, (e) 250, (f) 500, (g) 1000, (h) 2000 μM. (i) Ce^{4+}-ion-modified C-dots with scrambled DBA. (ii) Aptananozyme I. (iii) Aptananozyme II. (iv) Aptananozyme III. (v) Aptananozyme IV. (vi) Aptananozyme V. For all systems, the condition of the experiments corresponded to 0.8 μg mL^{-1}Aptananozymes, 5 mM H_2O_2.

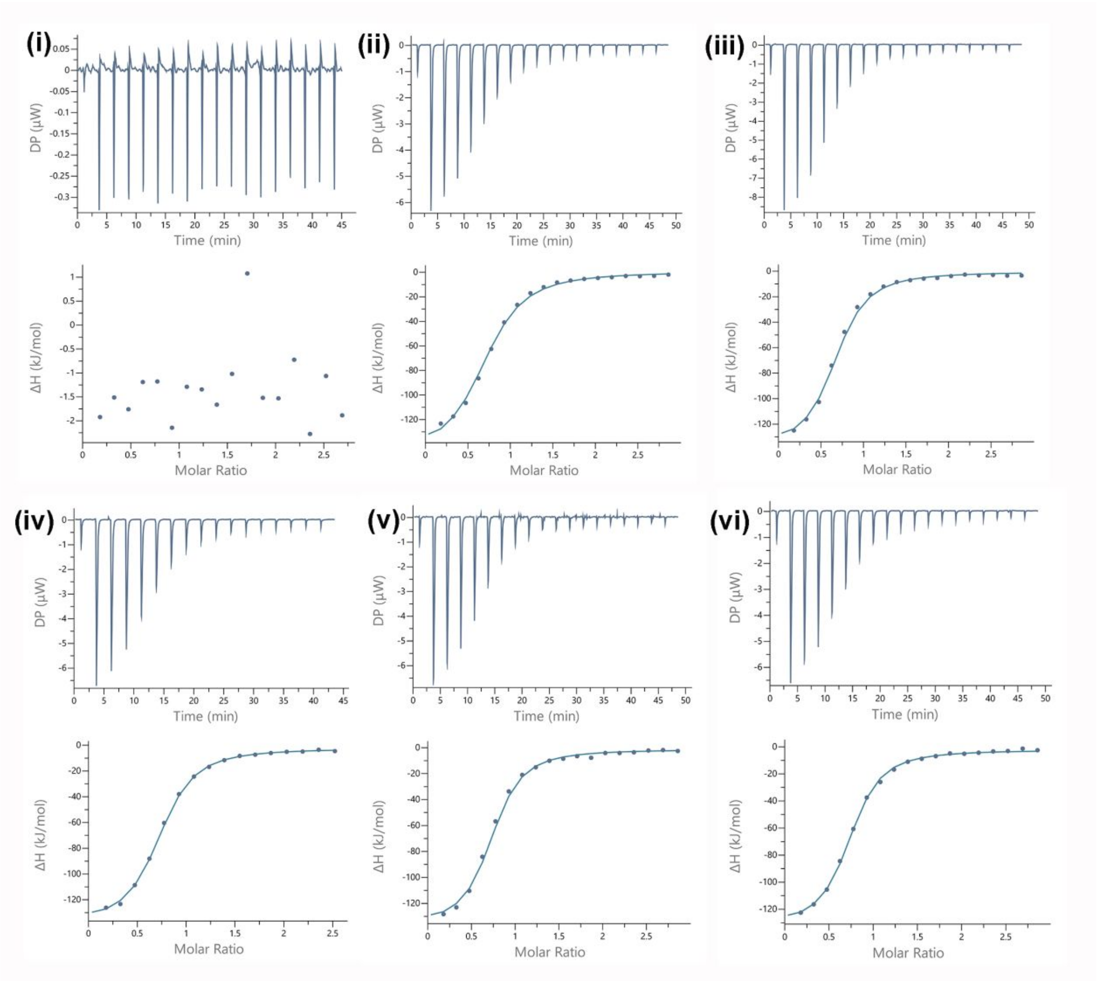

Figure S7. ITC plots (heat flow *vs* time and molar enthalpy change *vs* molar ratio) of different aptananozymes, with dopamine in 5 mM MES pH 5.5, 5 mM $MgCl_2$, 100 mM NaCl: (i) Ce^{4+}-ion-modified C-dots with scrambled DBA. (ii) aptananozyme I. (iii) aptananozyme II. (iv) aptananozyme III. (v) aptananozyme IV. (vi) aptananozyme V.

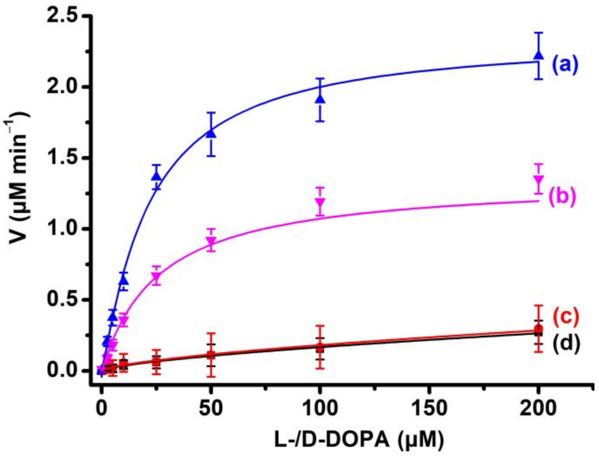

Figure S8. Rates corresponding to (a) the aptananozyme IV-catalyzed oxidation of L-DOPA by H_2O_2 to generate L-dopachrome in the presence of variable concentrations of L-DOPA and (b) the aptananozyme IV-catalyzed oxidation of D-DOPA by H_2O_2 to form D-dopachrome in the presence of variable concentrations of D-DOPA. (c, d) Rates corresponding to the oxidation of L-DOPA and D-DOPA in the presence of variable concentrations of L-/D-DOPA by H_2O_2, using the separated aptananozyme IV and the aptamer (**4**), respectively. Error bars are derived from $N = 3$ experiments.

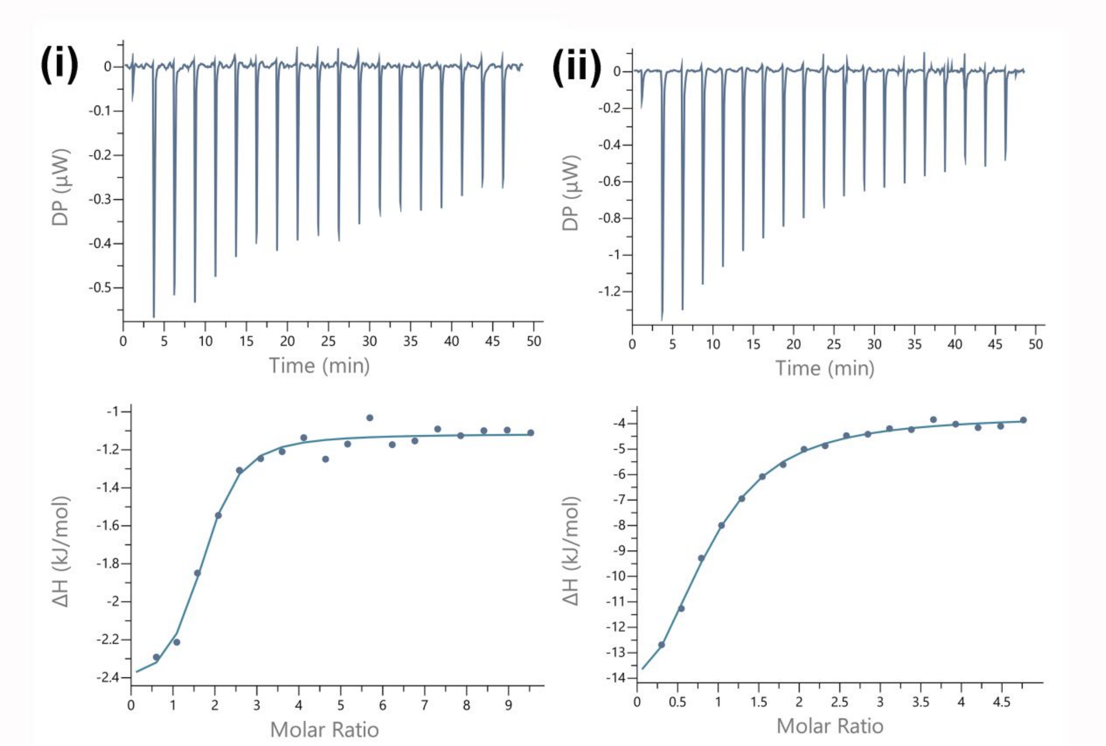

Figure S9. ITC plots (heat flow *vs* time and molar enthalpy change *vs* molar ratio) of aptananozyme IV, with L-DOPA (i) and D-DOPA (ii) in 5 mM MES (pH 5.5), 5 mM $MgCl_2$, 100 mM NaCl.

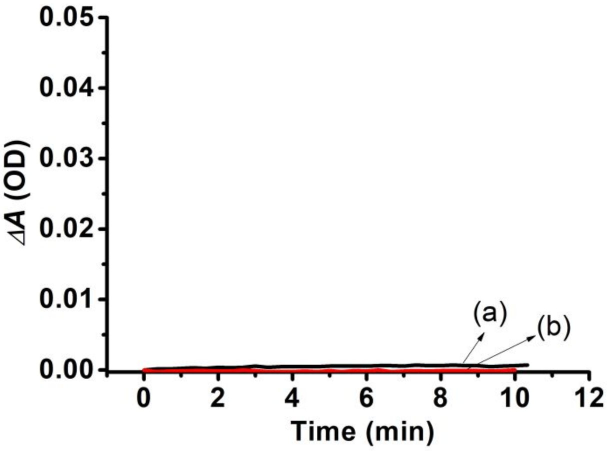

Figure S10. (a) Time-dependent absorbance of oxidation of dopamine by the “bare” non-Ce^{4+}-modified C-dots or the dopamine-aptamer modified C-dots in presence of H_2O_2.

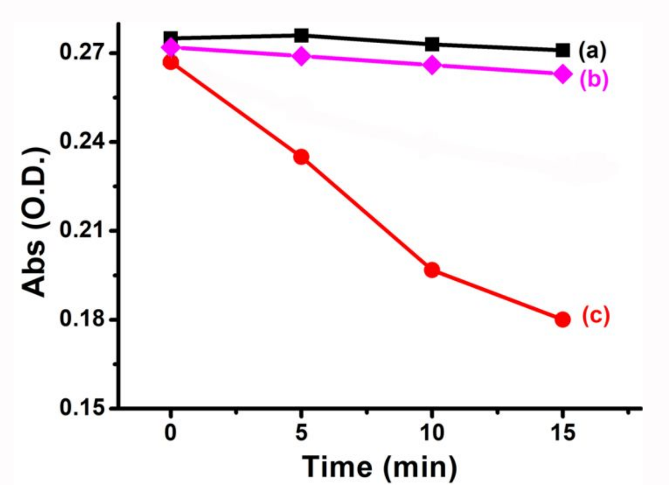

Figure S11. (A) Time-dependent absorbance changes of ROS agents (λ = 410 nm) in the systems composed of: (a) absence of H_2O_2, (b) presence of H_2O_2, (c) the AS1411-functionalized Ce^{4+}-ion-modified C-dots in the presence of H_2O_2.

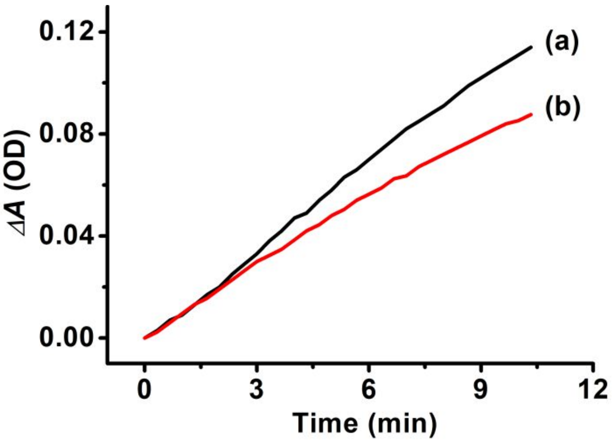

Figure S12. Time-dependent absorbance of oxidation of dopamine by the aptananozyme IV in presence of H_2O_2. Time-dependent absorbance of oxidation of dopamine by the aptananozyme IV incubated with cell lysis solution for 12 h, in presence of H_2O_2

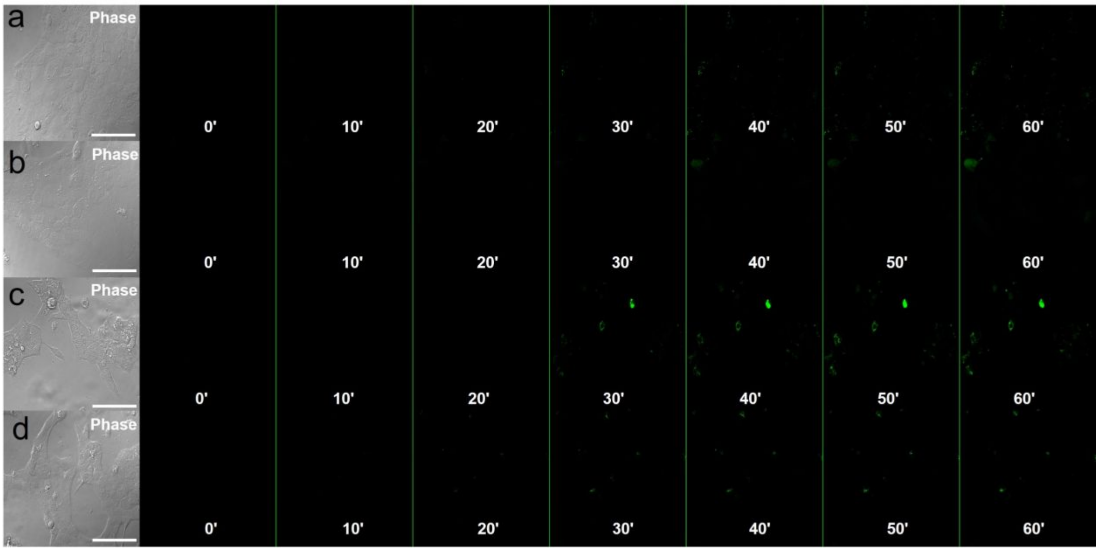

Figure S13. Confocal bright field and fluorescence images corresponding to the time-dependent formation of ROS intermediates probed by the C-DCDHF-DA dye in MCF-10A epithelial breast cells: (a) non-treated cells, (b) Ce^{4+}-ion-modified C-dots functionalized with a scrambled AS1411 aptamer sequence, (c) AS1411 aptamer-functionalized Ce^{4+}-ion-modified C-dots, (d) MUC1 aptamer-functionalized Ce^{4+}-ion-modified C-dots.

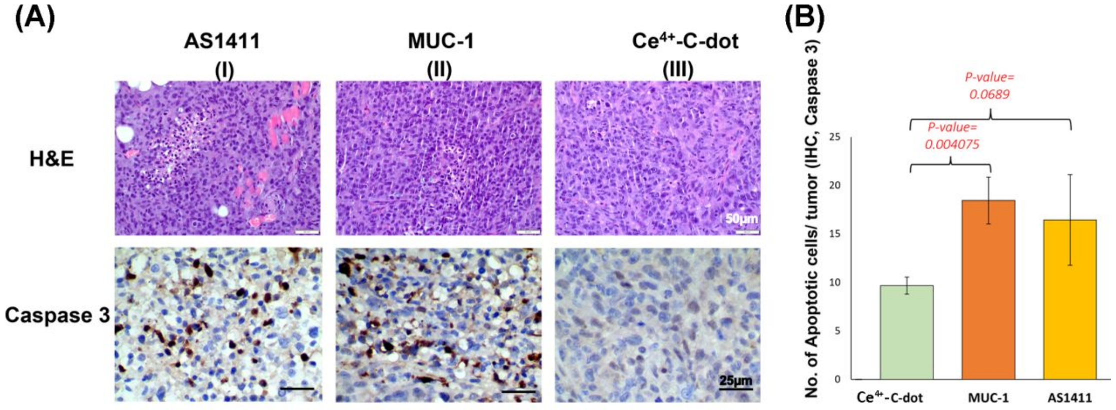

Figure 14. Histological analysis of the MDA-MB-231 xenografts treated with the Ce^{4+}-C-dots particles. (A) Histological analysis including hematoxylin and eosin (H&E) stained slides (Upper group) and Immunohistochemistry (IHC) (Bottom Group) against Caspase 3 the apoptosis marker, Panel I are the cells treated with AS1411, Panel II cells treated with MUC-1, Panel III control cells treated with Ce^{4+}- C-dotS. (B) Analysis of the IHC of caspase 3 positive apoptotic cells in all the tumors treated. All results were presented as mean ± SD. Significant results were evaluated using T-test (P-values presented on the figure).

Table S1. Concentration ratio of C-dots and DBA, and the loading of Ce^{4+}-ions on the C-dots, in different configuration of Aptananozymes.

Aptananozymes	*[C-dots]*:*[DBA]*	$m_{(C-dots)} : m_{Ce^{4+}}\ (mg)$
IV	1:5.1 ± 0.1	1:0.3 ± 0.001
III	1:5.3 ± 0.2	1:0.3 ± 0.002
I	1:5.2 ± 0.2	1:0.3 ± 0.002
II	1:5.3 ± 0.1	1:0.3 ± 0.002
V	1:5.0 ± 0.3	1:0.3 ± 0.001
Scrambled	1:5.1 ± 0.2	1:0.3 ± 0.001

Table S2. Kinetic parameters corresponding to the aptananozymes IV by using substrates of L-DOPA and D-DOPA.

Substrate	V_{max} (μM min^{-1})	K_M (μM)	k_{cat} (10^{-3} s^{-1})	K_d (μM)
L-DOPA	2.46±0.35	24.9±1.2	1.64±0.12	7.5±0.13
D-DOPA	1.35±0.32	26.1±5.1	0.86±0.02	2.6±0.15

Reference.

1. Sun, S.; Chen, Q.; Tang, Z.; Liu, C.; Li, Z.; Wu, A.; Lin, H. Tumor Microenvironment Stimuli-Responsive Fluorescence Imaging and Synergistic Cancer Therapy by Carbon-Dot-Cu^{2+} Nanoassemblies. *Angew. Chem., Int. Ed.* **2020**, *132*, 21227-21234.
